# In a comfort zone and beyond—Ecological plasticity of key marine mediators

**DOI:** 10.1002/ece3.6997

**Published:** 2020-11-10

**Authors:** Emilia Trudnowska, Kaja Balazy, Joanna Stoń‐Egiert, Irina Smolina, Thomas Brown, Marta Gluchowska

**Affiliations:** ^1^ Institute of Oceanology Polish Academy of Sciences Sopot Poland; ^2^ Nord University Bodø Norway; ^3^ The Scottish Association for Marine Science Oban UK

**Keywords:** Arctic, biological traits, *Calanus*, climate change, marine ecology, morphology, plasticity

## Abstract

Copepods of the genus *Calanus* are the key components of zooplankton. Understanding their response to a changing climate is crucial to predict the functioning of future warmer high‐latitude ecosystems. Although specific *Calanus* species are morphologically very similar, they have different life strategies and roles in ecosystems. In this study, *C. finmarchicus* and *C. glacialis* were thoroughly studied with regard to their plasticity in morphology and ecology both in their preferred original water mass (Atlantic vs. Arctic side of the Polar Front) and in suboptimal conditions (due to, e.g., temperature, turbidity, and competition in Hornsund fjord). Our observations show that “at the same place and time,” both species can reach different sizes, take on different pigmentation, be in different states of population development, utilize different reproductive versus lipid accumulation strategies, and thrive on different foods. Size was proven to be a very mutable morphological trait, especially with regard to reduced length of *C. glacialis*. Both species exhibited pronounced red pigmentation when inhabiting their preferred water mass. In other domains, *C. finmarchicus* individuals tended to be paler than *C. glacialis* individuals. Gonad maturation and population development indicated mixed reproductive strategies, although a surprisingly similar population age structure of the two co‐occurring species in the fjord was observed. Lipid accumulation was high and not species‐specific, and its variability was due to diet differences of the populations. According to the stable isotope composition, both species had a more herbivorous diatom‐based diet in their original water masses. While the diet of *C. glacialis* was rather consistent among the domains studied, *C. finmarchicus* exhibited much higher variability in its feeding history (based on lipid composition). Our results show that the plasticity of both *Calanus* species is indeed impressive and may be regulated differently, depending on whether they live in their “comfort zone” or beyond it.

## Graphical abstract

We are testing whether the morphological and ecological traits differ depending on the fact whether particular Calanus species (*C. finmarchicus* vs. *C. glacialis*) inhabit the preferable, original water mass (comfort zone) or exist in suboptimal conditions (due to, e.g., temperature, turbidity, competition). We show that both species can reach different sizes, take on different pigmentation, accomplish different state of population development, embrace different reproductive versus lipid accumulation strategies, and thrive on different food. This impressive plasticity differs depending if they live in their “comfort zone” or beyond it, which is of vital importance to understand the actual and to predict the future functionality of marine ecosystems.

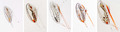

## 1 INTRODUCTION

The fundamental way by which organisms cope with climate change is through ecological plasticity, which encompasses any type of environmentally induced change (e.g., morphological, physiological, behavioral, phenological). Zooplankton can respond to global environmental changes phenotypically (with alterations in their physiology or behavior) or evolutionarily (with a shifting genetic composition of populations) (Dam, 2013; Kelly et al., 2017). It is still not known whether marine species will have the capacity to adjust to ongoing changes through phenotypic plasticity in the short term or adapt in the longer term (Byrne et al., 2020). Although ecological plasticity refers only to the phenotypic changes that are expressed in the lifetime of a single organism, some of these changes can potentially be adaptive (Ghalambor et al., 2007) and evolve over time and space (Kelly et al., 2017; Sasaki & Dam, 2019). Therefore, phenotypic plasticity plays a key role in adapting to new and changing environments (Chevin et al., 2010; Pfennig & Ehrenreich, 2014; Pfennig et al., 2010).

Copepods of the genus *Calanus* are the key components of zooplankton in the Arctic and northern Atlantic waters (Aarflot et al., 2017; Carstensen et al., 2019; Jaschnov, 1970), and they play a crucial role in marine food webs as the main mediators between the microbial system, phytoplankton, and higher trophic levels. Due to their high lipid content (Falk‐Petersen et al., 2009; van der Hoop et al., 2019; Mayzaud et al., 2016; Scott et al., 2000), they are responsible for the sustainability of large stocks of fish, seabirds, and marine mammals in the Arctic region (Falk‐Petersen et al., 2007, 2014; Stempniewicz et al., 2007). Additionally, due to the various centers of distribution of particular species, they are highly valued as biological indicators of the hydrographical–ecological regimes and consequently of the effects of ongoing climate changes (Choquet et al., 2017; Gabrielsen et al., 2012; Kwasniewski et al., 2010; Møller & Nielsen, 2019). Although specific *Calanus* species are morphologically very similar, they have different life strategies and exhibit different ecological traits, which at the same time are highly adaptable to the extremely variable environmental conditions encountered by *Calanus* copepods during their lifespan (Bandara et al., 2019; Falk‐Petersen et al., 2009; Feng et al., 2018; Kvile et al., 2018). Therefore, *Calanus* spp., owing to their high ecological plasticity, can reasonably be assumed to be able to cope with some extent with ongoing environmental changes (Byrne et al., 2020), but specific species may respond differently to warmer Arctic conditions (Falk‐Petersen et al., 2009; Kjellerup et al., 2012; Scott et al., 2000). However, due to problems with proper *Calanus* species identification, knowledge about their ecological plasticity and functioning in various oceanic conditions is still very limited.

In recent decades, the most common method for discriminating the two species was based on prosome length measurements (Arnkværn et al., 2005; Kwasniewski et al., 2003; Weydmann & Kwasniewski, 2008). However, this method was criticized recently because of an overlap in the size of the two species as an effect of their substantial size plasticity in response to food availability, water temperature, life cycle length, or even to predation pressure (Leinaas et al., 2016; Lindeque et al., 2006; Parent et al., 2011; Renaud et al., 2018). Genetic identification of *Calanus* species using either mitochondrial or nuclear markers (Leinaas et al., 2016; Lindeque et al., 2006; Parent et al., 2011; Renaud et al., 2018) was successful and offers a very promising, but still labor‐intensive, expensive, and rather unsuitable approach for large field surveys. The use of mitochondrial markers for the molecular separation between the two species has been challenged because of questions about possible hybridization between them in the northwest Atlantic (Parent et al., 2012), but subsequently developed nuclear markers for *Calanus* species identification (Smolina et al., 2014) can be used to detect events of potential recent hybridization (Nielsen et al., 2014). Nonetheless, large‐scale genetic surveys throughout the northeast Atlantic and Arctic oceans (Choquet et al., 2017, 2018) and novel studies of mitochondrial genomes of *C. finmarchicus* and *C. glacialis* (Weydmann et al., 2017) suggest that hybridization is unlikely. Consequently, all new studies clearly show that if *Calanus* is to be distinguished to the species level, this must be supported by genetic analysis (Choquet et al., 2018; Renaud et al., 2018). The proper identification of *Calanus* to the species level is definitely not only a matter of taxonomical curiosity; the knowledge of whether one or the other species prevails and what is the extent of its ecological plasticity is a principal ecological factor in understanding and predicting the future functionality of north polar marine ecosystems.

An increasing temperature and volume of Atlantic waters flowing into the Arctic is causing shifts in hydrographic conditions (Polyakov et al., 2005, 2020; Walczowski et al., 2012). Shifts in the positions of water masses will result in changes to the distribution of associated zooplankton communities (Beaugrand et al., 2002; Chust et al., 2014; Hays et al., 2005). Therefore, along with the progressive climate warming in the Arctic, a regime shift from larger lipid‐rich Arctic *C. glacialis* species toward smaller Atlantic *C. finmarchicus* species is expected (Aarflot et al., 2017; Kjellerup et al., 2012; Møller & Nielsen, 2019). Due to the commonly observed difference in the size and thus the amount of lipids between *C. finmarchicus* and *C. glacialis*, the two species were believed to support different arctic food webs (Renaud et al., 2018; Weslawski et al., 2009). The decreased availability of *C. glacialis* is expected to affect the breeding success of the most numerous seabirds, little auks (Jakubas et al., 2020; Stempniewicz et al., 2007). However, the predicted higher abundance of *C. finmarchicus* in the Arctic (Reygondeau & Beaugrand, 2011; Slagstad et al., 2011) is expected to favor fish stocks (Falk‐Petersen et al., 2007; Renaud et al., 2018; Stempniewicz et al., 2007). Additionally, the changes in the timing of *Calanus* reproduction and development rate have critical significance for their availability to predators (Balazy et al., 2019; Bandara et al., 2019; Søreide et al., 2008).

The aim of this study was to verify the actual differences between *C. finmarchicus* and *C. glacialis* with regard to their morphology (size, pigmentation), life cycle phenology (according to population demography and gonad maturation), and feeding strategies (variability in diet composition and differences in lipid accumulation). These differences were studied both in the waters where each species originate from (the Atlantic domain of the Polar Front in the case of *C. finmarchicus* and the Arctic domain of the Polar Front in the case of *C. glacialis*) and in the waters in which they coexist (Hornsund fjord, Spitsbergen), both in the fjord main basin and in its glacial bay. The hypothesis of this study is that the traits of the *Calanus* species (e.g., size, pigmentation, population demography, reproductive readiness, lipid accumulation, diet) will differ depending on whether specific species (*C. finmarchicus* vs. *C. glacialis*) inhabits the preferred original water mass (comfort zone) or exist in suboptimal conditions (due to, e.g., temperature, turbidity, competition). We assume that both species will exhibit high plasticity of the traits studied; however, the extent of the plasticity will differ between the species, with *C. finmarchicus* expected to be more of a generalist and *C. glacialis* expected to be more of a specialist (as defined by Dam, 2013). This study is the first to incorporate many aspects to improve taxonomical and ecological species recognition, which is the main prerequisite for predicting future ecosystem shifts in the Northern Hemisphere.

## 2 MATERIALS AND METHODS

**Figure 1:**
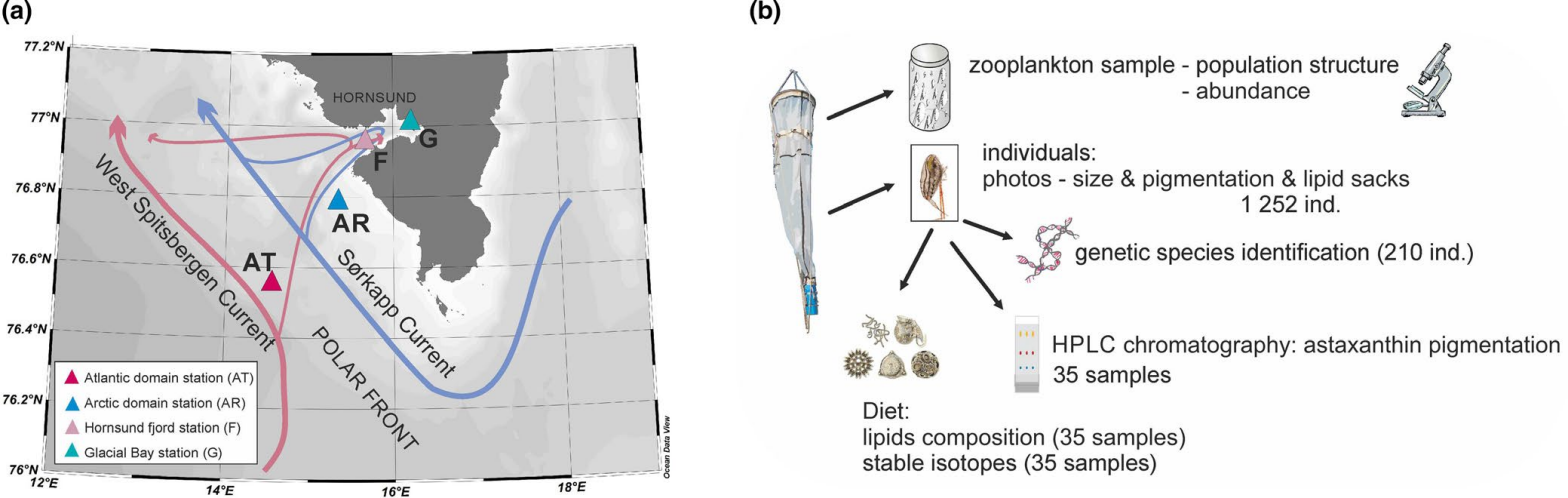
Sampling design. (a) Map of southern Spitsbergen showing the sampling stations located on both sides of the Polar Front and inside the Hornsund fjord. The arrows show the dominating ocean currents with their schematic path of advection to the fjord indicated by the narrower lines. (b) Scheme of the procedures with the collected material and splitting into sample categories

The study was performed in the Polar Front region in the southern part of the West Spitsbergen shelf in July 2018 (Figure 1). It was designed to collect *Calanus* either from the water mass they originate from (*C. finmarchicus* from the Atlantic Water (AT station) domain, from the West Spitsbergen Current that carries warm and saline Atlantic Water and *C. glacialis* from the colder and fresher Arctic‐type Sørkapp Current (AR station)), or from the Hornsund fjord, where two species co‐occur, both in the main fjord basin (F station) and in the glacial bay (G station) (Figure 1a). The station representing the Atlantic water domain (AT) was characterized by the highest water temperature, salinity, and chlorophyll fluorescence (Table 1). The lowest water temperature, salinity, and chlorophyll fluorescence were observed at the station located in the glacial bay (G). However, the glacial bay was characterized by extremely high concentrations of particles (Table 1).

**Table 1:** Mean and standard deviations (in upper right brackets) of environmental parameters averaged over upper 50‐m layer column at studied domains, AT (Atlantic), AR (Arctic), F (Fjord), and G (Glacial bay)

Region/ parameter	AT	AR	F	G
Temperature	7.4^(±0.3)^	3.7^(±0.6)^	3.1^(±0.2)^	2.2^(±0.2)^
Salinity	34.9^(±0.01)^	34.3^(±0.26)^	34.0^(±0.56)^	33.4^(±1.24)^
Chlorophyll fluorescence	0.53^(±0.2)^	0.32^(±0.1)^	0.35^(±0.3)^	0.11^(±0.1)^
Turbidity (particle concentration * 10^5^)	1.2^(±0.03)^	3.1^(±0.3)^	2.9^(±0.2)^	8.4^(±0.3)^

At each sampling station, two tows of a WP2 net (180 µm mesh size) were performed thorough the water column (70 m at G station, 200 m at F station, 170 m at AR station) or through the upper 200 m at the deep‐water AT station. The sample from the first haul of the net was immediately fixed in a formaldehyde–borax solution for the analysis of *Calanus* abundance, developmental stage composition, and gonad maturation stages. The samples from the next net hauls were put in buckets filled with seawater close to the in situ temperature. Then, the fifth copepodite stages of specific species (distinguished as small and large size modes) were photographed and preserved for further analyses (Table S1, Figure 1b).

Overall, 1,252 photographs of *Calanus* were taken and analyzed to determine the prosome length, the area of the lipid sac, and the pigmentation. Methods for specific morpho‐ecological traits were as follows:

### 2.1 Size and species identification verification

The prosome length of 1,252 photographed *Calanus* CV copepodites was measured in ImageJ/Fiji free software for image analyses from the tip of the cephalosome to the distal lateral end of the last thoracic somite. The medians and quartiles of prosome length measurements are presented as box plots. Additionally, 991 copepodites were measured from the formaldehyde‐fixed samples, among which 372 were CV copepodites, for which kernel density size distribution curves were fitted.

Genetic species identification was used to test the accuracy of the applied morphological method of species discrimination for further analyses. Genetic identification was performed for each individual using its antennae and a set of six nuclear insertion–deletion markers (InDels) in a multiplex polymerase chain reaction (PCR) following the protocol described in Choquet et al. (2017). Accuracy of the morphological identification was calculated as the percent between the number of correct assessments in relation to the number of all assessments. Overall, the accuracy was 93%, but the accuracy was also calculated separately for each species and sampling location (Table 2). The species discrimination was 100% accurate at the fjord station (F) and almost fully accurate at the AR and AT stations. In the Atlantic domain, 93% accuracy was obtained. In the Arctic domain, one individual with a size of 2.93 mm initially identified as *C. glacialis,* turned out to be *C. finmarchicus*. A high percent of the species misidentification (22%) occurred only in the glacial bay G station. All the *C. glacialis* were properly identified, but many of the small copepodites, assumed to represent C. *finmarchicus,* turned out to be small *C. glacialis,* with individuals as small as 2.39 mm.

**Table 2:** Accuracy of morphological species identification confirmed by genetics

Location	*C. finmarchicus*	*C. glacialis*	*N*
AT	0.93	–	30
AR	1	0.97	60
F	1	1	58
G	0.57	1	59

### 2.2 Red pigmentation

The quantification of astaxanthin content was based on high‐performance chromatography (HPLC) according to the methods described in Stoń‐Egiert & Kosakowska, 2005. Astaxanthin was isolated from previously lyophilized and weighed *Calanus* individuals by mechanical grinding in 90% acetone and sonication (2 min, 20 kHz, Cole Parmer, 4710 Series) for 2 hr in darkness. Then, after clarification, the extract was subjected to chromatographic analysis. The HP1200 system (Agilent, Perlan Technologies) was equipped with a C18 LichroCART™LiChrospher™ 100 RP18e (Merck) analytical column (dimensions 250 × 4 mm, particle size 5 μm, and pore size 100 Å). Pigments were separated in a gradient mixture of methanol, 1 M ammonium acetate, and acetone. Calibration was conducted with commercially available standards (The International Agency for 14C Determination DHI Institute for Water and Environment in Denmark), which allowed for the qualitative assessment of astaxanthin (based on retention time and similarity with the absorbance spectrum of the standards) and quantitative assessment (based on response factor values obtained during the calibration procedure).

Moreover, the pigmentation was analyzed by the visual examination of photographs taken of live individuals by a color coding scheme, depending on whether there was a full (>50%, red), mid (10%–50%, zebra), slight (<10%, piece), or no (0%, transparent) coloration of antennae and prosome. The photographs were also used to calculate the average pigmentation, where the rate of pigmentation was scored for each part of the body from 0 to 3 (e.g., antennae, prosome) and 0 to 1 (swimming legs, urosome) and summed, considering that approximately 10% of the pigmentation may be contributed by the urosome, 10% from the legs, 20% from the prosome, and 60% from the antennas.

### 2.3 Population development

The relative abundances of each of the *Calanus* copepodite stages were used to describe the population age structure. The abbreviations (CI‐AF) refer to six successive copepodite stages of *Calanus*, that is, CI, CII, CIII, CIV, and CV refer to the first five copepodite stages, and AF to adult females. The species identification of particular developmental stages was based on the size discrimination assessed for the populations in the Hornsund fjord (Weydmann & Kwasniewski, 2008). The age structure of specific species in the water domains studied was tested by Fisher's exact test in R (fisher.test(data)), an independence test to determine whether there is a significant relationship between two categorical variables.

The gonad maturation stages of *Calanus* females were determined as described by Niehoff (2007) and Niehoff and Runge (2003). In our case, we distinguished stage G3, characterizing females that are preparing for spawning (multiple layers of oocytes in both anterior and posterior diverticula); stage G3.5, indicating females that are ready to spawn (ventral layer of oocytes is already colored, but not yet fully brown); and G4, representing females that would spawn within hours (oocytes undergoing final maturation in the most ventral layer in the gonads). The spent stage (S) characterizes females that had already finished reproduction.

### 2.4 Lipid content

Lipid content was determined as the mass of lipids that was extracted for the fatty acid composition analyses and estimated by the measurements of the lipid sac area. The lipid sac area was manually measured by contouring the sac perimeter by hand in all the photographed copepods. Furthermore, the specific equations derived from the reliable calibration of the individual lipid contents of Arctic copepods (Vogedes et al., 2010) were applied to calculate the total lipid content. Then, the percentage of the lipid sac area (fulfillment) was calculated as a function of the total area of the prosome. To verify whether the lipid content differed between the two species, the unpaired two‐sample Mann–Whitney test was performed, and to verify whether the lipid content differed among study locations and species, the nonparametric Kruskal–Wallis test was performed in R (Package “stats”). The output of the Kruskal–Wallis test indicated whether there is a significant difference between groups, but to know which pairs of groups are different, the function pairwise.wilcox.test was used to calculate pairwise comparisons between groups.

### 2.5 Diet

To verify the source of stored lipids in of the two species occurring at the same time in diverse water masses and in fjord waters, where they coexist, a combination of stable isotope (δ^13^C, δ^15^N) and fatty acid composition analyses was performed.

Stable isotope analysis was performed following the protocol of Lebreton et al., (2012). Samples were analyzed using an elemental analyzer (Flash EA 1112, Thermo Scientific, Milan, Italy) coupled to an isotope ratio mass spectrometer (Delta V Advantage with a ConFLo IV interface, Thermo Scientific, Bremen, Germany). The results are expressed in the δ unit notation as deviations from standards (Vienna Pee Dee Belemnite for δ13C and N2 in air for δ15N) following the formula: δ^13^C or δ^15^N = [(R_sample_/R_standard_) − 1] × 103, where R is 13C/12C or 15N/14N, respectively. Calibration was performed using reference materials (USGS‐24, IAEA‐CH6, IAEA‐600, USGS‐61, and USGS‐62 for carbon; IAEA‐N2, IAEA‐NO‐3, IAEA‐600, USGS‐61, and USGS‐62 for nitrogen). Analytical precision based on the analyses of acetanilide (Thermo Scientific) used as laboratory internal standard was \0.1 and \0.15 ‰ for carbon and nitrogen, respectively.

The identification and quantification of fatty acid methyl esters (FAMEs) were determined by gas chromatography/mass spectrometry (GC/MS) according to the method of Brown et al. (2011). An internal standard (10 μl; 1 mg/ml nonadecanoic acid) was added to lyophilized and weighed *Calanus* samples. Samples were then saponified (20% KOH; 70°C; 60 min). Fatty acids were obtained by the addition of concentrated HCl (0.5 ml) to the saponified solutions followed by extraction into hexane (3 × 1 ml). Fatty acids were then methylated (1 ml; 1:9 HCl:MeOH; 80°C; 60 min) and re‐extracted in hexane (3 × 1 ml) prior to analysis (Shimadzu QP2010 gas chromatograph coupled to a QP2020 quadrupole EI mass spectrometer; HP5ms). FAMEs were identified by comparison to authenticated standards (Supelco 37 Component FAME Mix), retention times, and mass spectral library matches (>95% confidence). Instrumental abundances were normalized to the internal standard and sample mass for quantification. The interpretation of the lipid tracers is based on the review by Lee et al. (2006). The differences in the composition of fatty acids were tested by the PermANOVA statistics, with fixed factors of species and water domain. A nonparametric multivariate, permutational ANOVA was used to compare groups of objects and test the null hypothesis that the centroids and dispersion of the groups as defined by the measured space are equivalent for all groups. The calculations were performed in PRIMER v7 and PERMANOVA software.

## 3 RESULTS

### 3.1 Size

**Figure 2:**
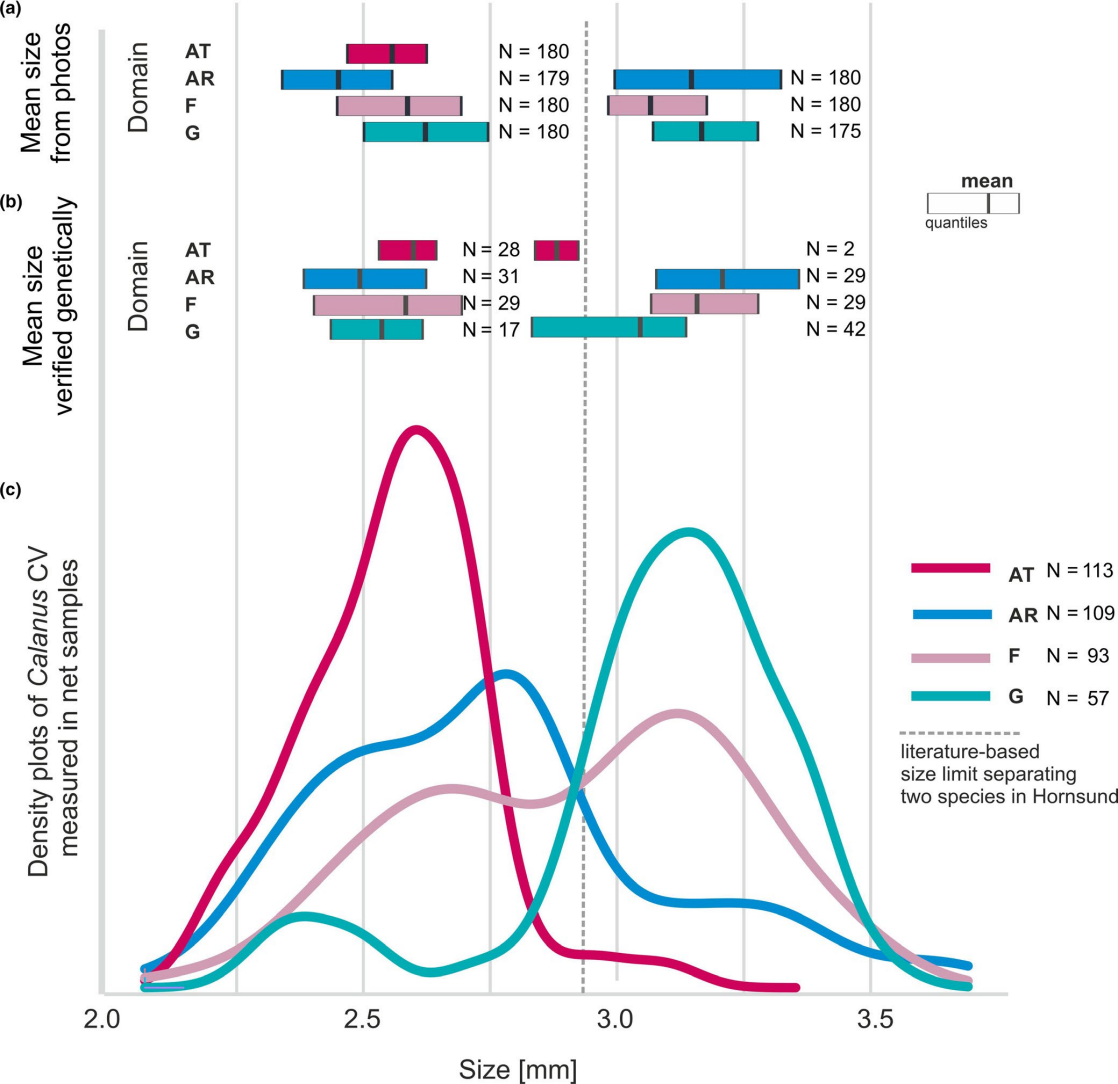
(a) Box plots of the mean size (prosome length measured from the photographs of alive individuals) of the *Calanus* CV that were selected for further analyses, basing on the two size modes representing the two species: smaller *C. finmarchicus* and larger *C. glacialis*. (b) Box plots of the mean size, with *Calanus* CV species tested genetically. C) Density plots of the prosome length of *Calanus* CV from the preserved samples

A clear unimodal size distribution of *Calanus* copepods from the AT domain (Figure 2c), with an average prosome length of 2.6 mm, enabled the easy selection of *C. finmarchicus* for further analyses (Figure 2a). At the AT station, all copepods were assumed to be *C. finmarchicus,* but two large (2.79, 2.96 mm) individuals turned out to be *C. glacialis* (Figure 2b). Three size modes of *Calanus* were distinguished in the AR domain (Figure 2c). For further data analyses, two clear size modes of *Calanus* were selected at the AR station as representative of the two species, that is, the smallest (approximately 2.47 mm), assigned as *C. finmarchicus,* and the largest, albeit least dominating (app. 3.16 mm), size mode was assigned as *C. glacialis* (Figure 2a). At station F, two distinct size modes (2.6 and 3.1 mm) clearly represented specific species (Figure 2aBC). In the glacial bay, *C. glacialis* was found to be either large (3.1 mm) or much smaller than the assumed size limit (2.4–2.9, on average 2.7 mm), so the samples assigned as *C. finmarchicus* at station G (Figure 2a) were substantially (approximately 40%) contaminated by the smaller sized *C. glacialis* (Table 1).

### 3.2 Pigmentation

**Figure 3:**
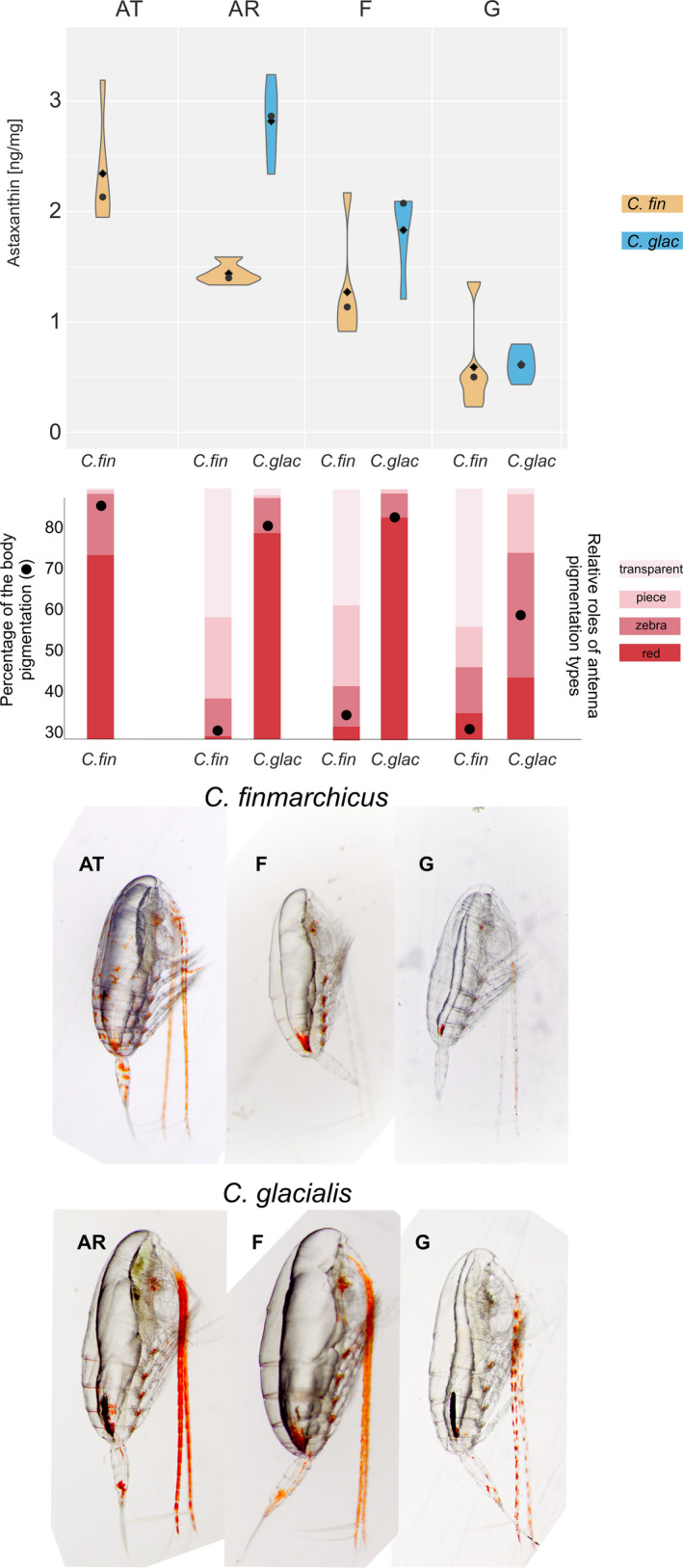
*Calanus* pigmentation in relation to sample origin. The violin plots present the mean (dot) and median (diamonds) concentrations of the astaxanthin pigment in the *Calanus* copepods sampled in various water domains (AT—Atlantic, AR—Arctic, F—fjord, G—glacial bay). Bar plots present the different types of antenna pigmentation. Dots present the estimated percentage of the red pigmentation of the *Calanus* body, calculated based on antenna, prosome, swimming leg, and urosome coloration)

The amount of astaxanthin pigment was the highest in the *Calanus* copepods inhabiting their original water mass (*C. finmarchicus* at the AT station and *C. glacialis* at the AR station) (Figure 3). In both species, the amount of this red pigment decreased progressively from the open water station toward the inner fjord waters, with the intermediate concentrations of astaxanthin in the fjord (F station) and the lowest concentrations observed in the glacial bay (G station).

The majority of the *C. finmarchicus* specimens had very red antennae in their original water mass (AT), and antennae were rather pale/transparent for individuals on the shelf (AR) and in the fjord (F) (Figure 3).*C. glacialis* had red antennae not only in their original water mass (AR) but also in the fjord (F) main basin, and they had striped, “zebra style” antennae, in the glacial bay (G). A similar trend of red coloration was also observed for all body parts, which were highly pigmented (>80%) in *C. finmarchicus* at the AT station and in *C. glacialis* at the AR and F stations (Figure 3).

### 3.3 Population development

**Figure 4:**
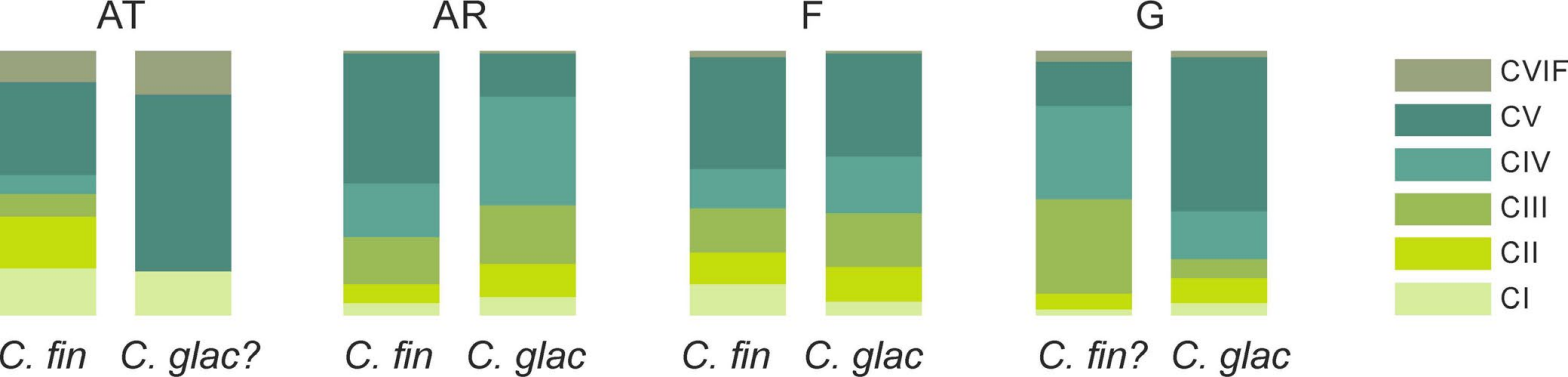
The relative proportions of various life stages (CI—CVIF) of specific *Calanus* species in specific water domains (“?” is used when the species identification by size is uncertain)

The age structure of *C. finmarchicus* at the AT station did not differ significantly from the demographic structure found on the shelf (AR) and in the fjord (F) (Fisher's exact test, *p* < .001). However, approximately half of the population was represented by the CV life stage at the AR and F stations, while a high proportion of females and the first copepodite stages was found at the AT station (Figure 4). Additionally, the relative proportions of the three youngest life stages of *C. glacialis* were similar between the shelf (AR) and the fjord (F); however, the proportion of CV relative to CIV was higher in the fjord. The demographic structure of *C. glacialis* was advanced in the glacial bay (G) but was not significantly different from the structure in the fjord (Fisher's exact test, *p* < .001). Surprisingly, the greatest degree of synchronization in population development between the two species was observed in the fjord (Fisher's exact test, *p* = .36).

**Figure 5:**
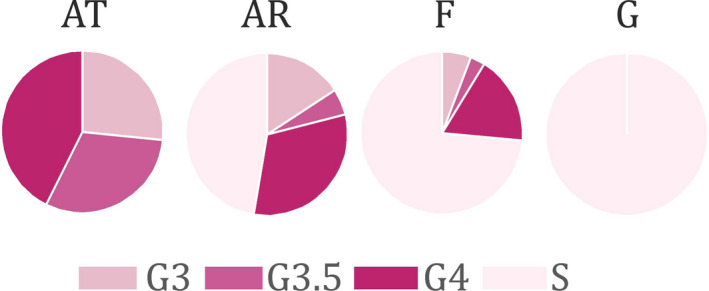
The relative proportions of various maturation stages of female gonads in specific water domains

The analysis of the gonad maturation stages indicated that the females were either almost ready to spawn (G3 and G3.5), actively spawning (G4), or they had finished reproduction (S). All females actively produced eggs at the AT station (Figure 5), half of them on the shelf (AR station) and only a quarter in the fjord (F station). In the glacial bay (G station), there were only a few females, and none of them reproduced (Figure 5).

### 3.4 Lipid content

**Figure 6:**
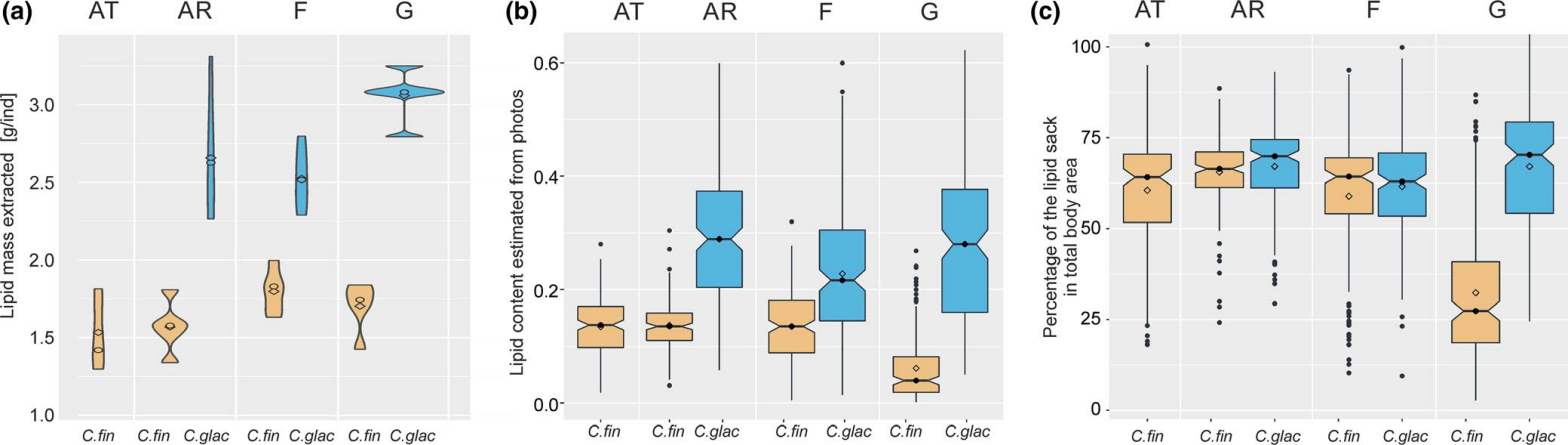
The lipid content for *Calanus* in various water domains (AT—Atlantic, AR—Arctic, F—fjord, G—glacial bay). (a) Violin plot of the amount of lipids extracted. (b) Box plots of the lipid content estimated from the lipid sack measurements from photographs. (c) The percentage of lipids in the body area. Speciefic species marked with colours: *C. finmarchicus* (yellow) and *C. glacilias* (blue)

Because of the larger body size of *C. glacialis*, the total amount of lipids per individual, either extracted or measured from photographs, was higher in *C. glacialis* than in *C. finmarchicus* in each of the oceanographic domains (Mann–Whitney U tests, *p* < .001) (Figure 6). The lipid content and the percentage of lipids in total body area were lower at F station than at AR and G stations in the case of *C. glacialis* (Kruskal–Wallis test with pairwise comparisons using Wilcoxon tests, *p* < .001). The lipid content of *C. finmarchicus* was lower at G station than at the other stations (Kruskal–Wallis test with pairwise comparisons using Wilcoxon tests, *p* < .001). The fulfillment of copepods (calculated as the percentage volume area of the lipid sack in comparison with the total body volume) differed for *C. finmarchicus* among stations, with the highest lipid fulfillment at the AR station, a lower fulfillment observed in an important group of individuals at the AT and F stations, and the lowest lipid fulfillment at the G station (Kruskal–Wallis test with pairwise comparisons using Wilcoxon tests, *p* < .001).

### 3.5 Diet

**Figure 7:**
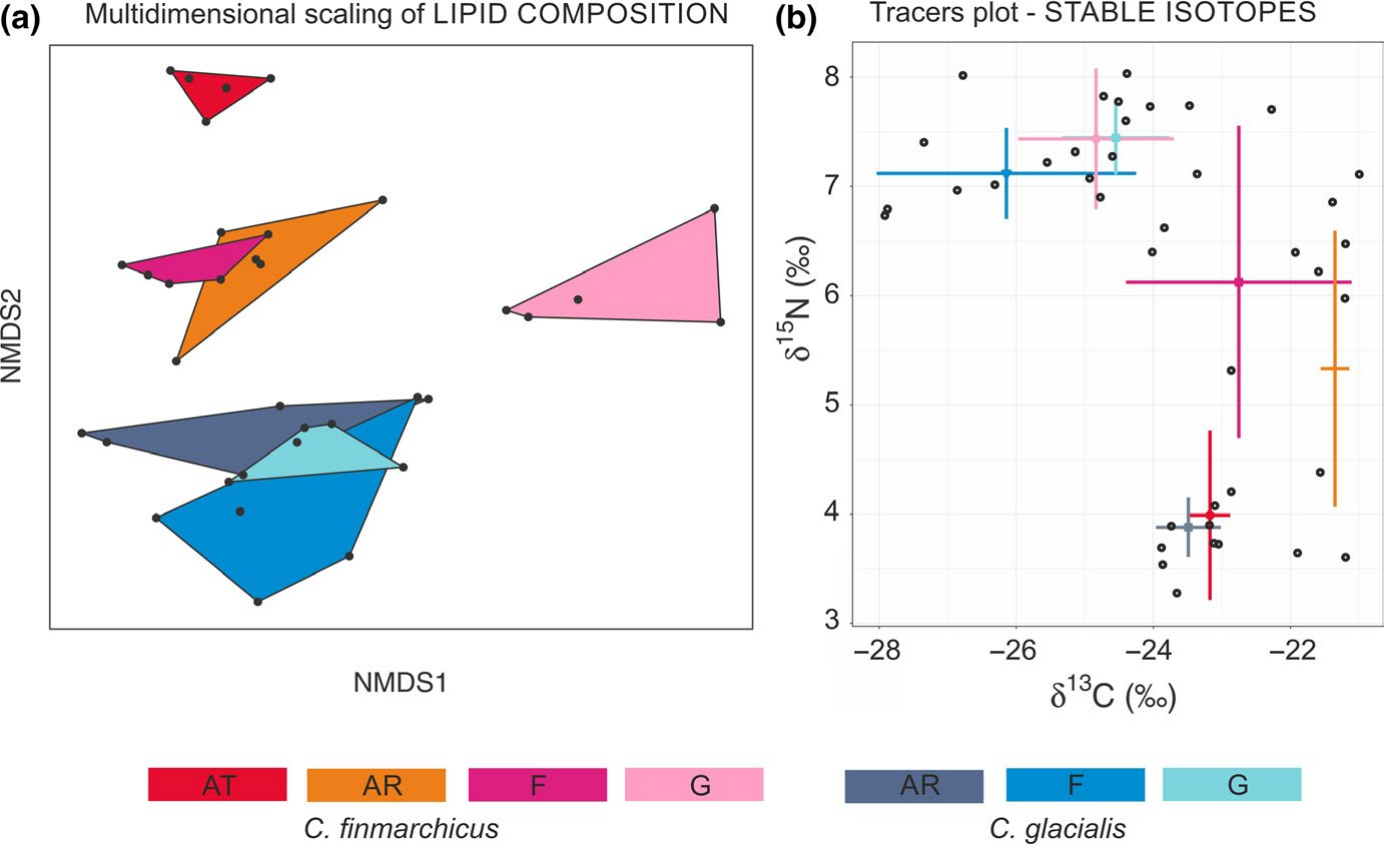
(a) The multidimensional scaling of the *Calanus* lipid composition based on the 20 classes of the fatty acids. (b) The isotopic composition is presented on the trace plot, as an output of stable isotope mixing model (“simmr” library in R). Colors refer to specific species in specific studied domains (AT—Atlantic, AR—Arctic, F—fjord, G—glacial bay)

The fatty acid composition of lipids was similar in *C. glacialis* individuals (Figure 7a), regardless of the water domain (PermANOVA, *p* > .05). The composition of fatty acids was different between the two species (PermANOVA, *p* < .001). *C. finmarchicus* had a similar composition of fatty acids on the shelf (AR) and in the fjord (F) (PermANOVA, *p* > .05), but compositions were different in the core of the Atlantic water domain (AT) and in the glacial bay (G) (PermANOVAs, *p* < .05) (Figure 7a). The long‐chain highly energetic C20‐22 monounsaturated fatty acids (MUFAs) dominated in both species, constituting 43% (38%–48%) in *C. glacialis* and 41% (36%–45%) in *C. finmarchicus* (Tables S1, S2). The highest percentage of the diatom fatty acid marker 20:5(n‐3) (eicosapentaenoic acid; EPA) was observed in *C. finmarchicus* at AT station and in *C. glacialis* at AR and G stations (13%). A high percentage of the dinoflagellate marker 22:6(n3) (docosahexaenoic acid; DHA) was observed in *C. finmarchicus* at AT station (10%), while the lowest percentage was observed at F station (7%). In *C. glacialis,* DHA contributed approximately 7% to the total lipid composition, with only a slightly higher contribution at F station (8%). The DHA/EPA ratio was lower than 1 in both species, suggesting that diet was more diatom‐based than flagellate‐based, especially in *C. glacialis* at the G and AR stations and in *C. finmarchicus* at the AT station (0.6). In all domains studied, *C. glacialis* was also rich in C18 PUFAs (18%), especially 18:4(n‐3), which is also important in the haptophyceae *Phaeocystis pouchetii*, whereas the amount of this acid was elevated in *C. finmarchicus* only on the shelf (AR) (16%). The fatty acid related to an omnivorous diet (18:1n9) was especially high in *C. glacialis* at the F station (12%) and the lowest in *C. finmarchicus* at the AT and F stations. The amount of 18:2n6, regarded as a tracer of freshwater/terrestrial input, was generally very low (less than 0.5%) (Tables S1, S2).

According to the ratios of stable isotopes, the lowest δ^15^N values were recorded in both *Calanus* species in their original water masses (in *C. finmarchicus* at the AT station and in *C. glacialis* at the AR station) (Figure 7b). The median and most variable values of δ^15^N were found in *C. finmarchicus* in the Arctic domain (AR) and inside the fjord (F). The highest δ^15^N, associated with the lowest δ^13^C, was recorded in *C. glacialis* in the fjord, both in the main basin (F) and in the glacial bay (G), which was very similar in the small *Calanus* size fraction at the G station.

## 4 DISCUSSION

Many organismal traits exhibit phenotypic plasticity in response to environmental variation. Therefore, it is crucial to provide ground truth data on possible plasticity in the basic morphological and ecological traits of the key high‐latitude zooplankton species expected to make up a new Arctic regime in different ways. Such knowledge would be of great importance to verify and extend many existing models aimed at predicting the future of the Arctic ecosystem based on *Calanus* ecology (Banas et al., 2016; Bandara et al., 2019; Feng et al., 2018; Ji et al., 2012; Melle et al., 2014; Renaud et al., 2018).

To be able to pinpoint differences in ecological plasticity, it is important to know whether individuals from studied locations represent a panmictic unit or genetically distant units (then observed differences could rather be due to local adaptation). Despite extensive research effort, population genetic differentiation in *Calanus finmarchicus* and *C. glacialis* is still under debate with conclusions ranging from a lack of population genetic structure in the North Atlantic for *C. finmarchicus* (Provan et al., 2009) and in the Arctic for *C. glacialis* (Weydmann et al., 2016) to a large‐scale structure in the North Atlantic for *C. finmarchicus* (Unal & Bucklin, 2010) and between the Pacific and the Arctic Oceans for *C. glacialis* (Nelson et al., 2009). Preliminary results from recent advances in *Calanus* genomics using a capture enrichment‐based approach in three population along the Norwegian coast showed weak or lack of genetic structure in *C. finmarchicus* and stronger genetic structure in *C. glacialis* (Choquet et al., 2019). Although more populations need to be examined using such genome‐wide approach for a conclusion on *Calanus* population genetic structure, it is reasonable to assume that the small‐scale sampling locations in this study belong to a panmictic population for both species, while the term “population” in further discussion refers to a group of individuals that developed at same location, and therefore, same conditions.

### 4.1 Size

For decades the species identification between *C. finmarchicus* and *C. glacialis* based on size separation was a convenient simplification, and according to this study, it is still a reliable method in the studied fjord, where two distinct size modes agreed well with previously recognized size ranges of both species (Weydmann & Kwasniewski, 2008). However, such size‐based identification has been shown to be unreliable in some areas (Choquet et al., 2018; Gabrielsen et al., 2012; Lindeque et al., 2006; Renaud et al., 2018), which was also the case in our study of shelf and glacial regimes. *C. glacialis* individuals were occasionally observed to be smaller than assumed. Size reduction in *C. glacialis* has frequently been recorded and mostly explained by the considerable plasticity of this species in relation to temperature differences (Choquet et al., 2018; Leinaas et al., 2016), the shorter length of its life cycle (Gabrielsen et al., 2012), or even by predator pressure (Bandara et al., 2019; Berge et al., 2012). However, assuming that in turbid glacial waters, their visual susceptibility to predation is already greatly reduced, the size reduction in *C*. *glacialis* should rather be considered as an effect of suboptimal living conditions, including extreme hydrographic dynamics and worsened food quality.

Unfortunately, the most intriguing, trimodal size distribution of *Calanus* observed on the Arctic side of the Polar Front (AR station) was not tested thoroughly, and only the two ends of the size spectrum were investigated further. Therefore, it remains an open question whether the numerically dominating medium‐size mode (2.75 mm) was represented by small *C. glacialis* or by large *C. finmarchicus*. The prevailing abundance of the smaller size mode of *Calanus* at this station could suggest that advection of the Atlantic water across the Polar Front is higher than we assumed and thus leads to the predomination of *C. finmarchicus*. However, high size variability of genetically proven *C. glacialis* was observed at this station, so instead, we suggest that this mid‐size fraction could represent a population of *C. glacialis* coming from the northern Barents Sea.

Interestingly, a few large individuals, proved genetically to be *C. glacialis,* were also found in the Atlantic sector of the Polar Front (AT station). Additionally, Basedow et al. (2019) observed singular individuals of *C. glacialis* offshore from the northern Norwegian coast, which implies that the fjord and shelf populations of *C. glacialis* can be transported far offshore over the shelf edge and thus can be mixed with *C. finmarchicus* even within the core of its northward flowing Atlantic water current.

### 4.2 Pigmentation

In line with our expectations, both species exhibited pronounced red pigmentation when inhabiting their preferred water mass (the Atlantic domain of the Polar Front in the case of *C. finmarchicus* and the Arctic domain of the Polar Front in the case of *C. glacialis*). This observation, supported by the recent test of various morphological traits of *Calanus* species identification methods (Choquet et al., 2018), suggests that the red pigmentation of the antenna is not a species‐specific feature, as was proposed by Nielsen et al. (2014). The red pigmentation of *Calanus* appears to instead be location‐specific. Following our hypothesis about the “comfort zone,” the red pigmentation of *C. glacialis* in Hornsund suggests that the environmental conditions in the fjord are more similar to the Arctic and preferable for *C. glacialis,* which agrees with its general dominance in the Hornsund fjord (Gluchowska et al., 2016; Trudnowska et al., 2014; Weydmann & Kwasniewski, 2008). In the Arctic, *C. finmarchicus* individuals tend to be paler compared with *C. glacialis,* either in Svalbard (Choquet et al., 2018) or in Greenland (Nielsen et al., 2014).

However, which species synthesizes this carotenoid pigment and at what intensity to protect the accumulated lipids from oxygenation are also strongly dependent on many factors other than water mass, including the depth of their occurrence and their diet (Mojib et al., 2014; Sommer et al., 2006). In this case, the vertical position of copepods could indeed play a role, as *C. finmarchicus* most likely persists in intermediate water layers in the fjord, where Atlantic water is advected to the Hornsund (Promińska et al., 2018). This deeper occurrence may explain their reduced need for protection against UVR irradiance in the fjord in contrast to the increased photoprotection needed in the offshore Atlantic waters, where *C. finmarchicus* tends to concentrate in the upper few meters (Basedow et al., 2019; Trudnowska et al., 2015).

Copepods can adjust their level of red pigmentation quickly, even within a season, depending on the prevailing threat, UVR, predators (Hansson, 2000), or parasites. According to our observations, they can switch from red to pale within hour (e.g., when in a bucket in the fridge without any light), but they can become again red almost immediately when exposed to a light source. However, not all of individuals could return red pigmentation, even after long exposure to light. The fact that some individuals stayed pale may be explained by their need to have special precursors of carotenoids in their diet (Mojib et al., 2014; Sommer et al., 2006). Indeed, both species had similar stable isotope compositions in their “comfort zones” (both exhibiting high levels of red pigmentation) and variable stable isotope and lipid compositions, where their redness was variable.

### 4.3 Population development

The observed synchronization in the population age structure between the two species in the fjord was surprising in light of the documented differences in reproductive timing and strategies of specific *Calanus* species (Falk‐Petersen et al., 2009; Søreide et al., 2010), which is observed even when they co‐occur in the same region (Arnkværn et al., 2005; Niehoff et al., 2002; Swalethorp et al., 2011). The similar age structures of both species may indicate that favorable feeding conditions occurred in the fjord prior to our sampling, which enabled both species to simultaneously develop and to adopt a similar strategy. Additionally, the transport of copepodites from the shelf could play a role, as the *Calanus* age structure in fjords is frequently found to be related to various advection rates (Kwasniewski et al., 2003; Trudnowska et al., 2020; Willis et al., 2006). This could be especially the case for *C. finmarchicus*, whose demography was very similar between the fjord and shelf and which is probably mostly advected to the fjord. The small‐scale difference between fjord and shelf populations in demographic structure of *C. glacialis,* especially regarding the relative proportions between younger life stages and the most lipid‐rich fifth stage (CV), could be of great importance for CV‐selective predators such as little auks (Balazy et al., 2019; Jakubas et al., 2020; Welcker et al., 2009) and may explain why birds tend to forage on the shelf rather than in the fjord in the time window of their main feeding requirements (Jakubas et al., 2013).

Actively reproducing *C. finmarchicus* in Atlantic waters, in contrast to less active reproduction in the Hornsund fjord and shelf populations, which seemed to adopt the lipid accumulation strategy more, is possibly the other indication that the reproductive strategies of *Calanus* are very plastic (Daase et al., 2013; Kvile et al., 2018). Most likely mixed reproductive strategies will enable *Calanus* to actively and flexibly optimize their growth and development to adapt to the expected variability in the timing and quality of the pulses of primary production (Ejsmond et al., 2018; Ji et al., 2013).

### 4.4 Lipids

One of the key and most spectacular features of *Calanus* is its ability to accumulate large lipid reserves during a short grazing season in late spring and summer (Daase et al., 2013; Falk‐Petersen, et al., 2007; Søreide et al., 2010; Wassmann, 2011). In our study, in most cases copepods were very full of lipids (approximately 60% of the body area was filled by the lipid sack), which means that they were well into the lipid accumulation process for a winter diapause phase. The lipid content and lipid fulfillment were highly variable, indicating strong interindividual differences. Moreover, lipid accumulation was not species‐specific, which agrees perfectly with the recent study by Renaud et al. (2018). Interestingly, no correlation was found between red pigmentation and the lipid content or the percentage of lipids in the total body area (Pearson correlation, *p* > .005).

In the glacial bay, individuals of the large *Calanus* size mode were extremely full of lipids in contrast to ones from the small size mode, in which very small lipid sacks were observed. Whereas, in the fjord, *C. glacialis* exhibited decreased lipid accumulation perhaps as an effect of the competitive regime with *C. finmarchicus* or because of the different niche partitioning, which is in line with the different lipid and stable isotope compositions of both species in the fjord.

### 4.5 Diet

Traditionally, *Calanus* copepods have been regarded as herbivores; however, their omnivore preferences (Cleary et al., 2017; Levinsen et al., 2000; Søreide et al., 2008; Yeh et al., 2020) and their ability to adjust to shifts in the timing and availability of their prey (Banas et al., 2016; Forest et al., 2011; Freese et al., 2016) can vary greatly. In addition, the complex hydrography (i.e., mixing of Atlantic, Arctic, and coastal water masses) around Svalbard significantly expands the pool of resources and niche diversity in terms of their habitat and diet (Kortsch et al., 2019). According to the δ^15^N stable isotope and lipid composition, both species had a mostly herbivorous, diatom‐based diet in their original water masses (*C. finmarchicus* at the AT station and *C. glacialis* at the AR station).

However, the overall lipid composition differed significantly between the two species, regardless of the region. The differences in the feeding patterns of *Calanus* were found not only to be species‐specific (Scott, Kwasniewski, Falk‐Petersen, & Sargent, 2000, 2002), but also to be related to seasonal and environmental changes (Forest et al., 2011; Mayzaud et al., 2016; Melle et al., 2014; Yeh et al., 2020). Such striking differences in the diets of the co‐occurring species is a question to consider whenever we deal with the advected versus local populations or if they are occupying different water layers and thus are exposed to various food sources. Such water column partitioning in utilizing different available resources by grazing at different depths was recently explained as a way to minimize the competition between the two *Calanus* species (Schmid & Fortier, 2019). Additionally, Trudnowska et al. (2015) observed the bimodal vertical separation of *Calanus* spp. on the west Spitsbergen shelf, both between the species and individuals at different life stages.

While the diet of *C. glacialis* was rather similar among the studied domains, just with a more herbivorous tendency on the shelf and a slightly more omnivorous tendency in the fjord, *C. finmarchicus* exhibited much higher variability and differences in its feeding history. The populations found in the fjord and on the shelf showed elevated concentrations of the marker of *Phaeocystis* flagellates (Leu et al., 2006), which is indeed often dominant in the Svalbard fjords during summer (Kubiszyn et al., 2017; Piwosz et al., 2009).

As expected, the copepods in the glacial bay were depleted in ^13^C, which is a signature of the terrestrial input to their diet (Dalsgaard et al., 2003) or starvation (Mayor et al., 2011). Additionally, in the photographs, we could see that they were loaded with mineral particles. The more omnivorous diet of both *Calanus* species in the glacial bay (highest δ^15^N) was probably caused by the decreased availability of phytoplankton due to diminished light conditions and the higher importance of microzooplankton in turbid waters (Halbach et al., 2019; Piwosz et al., 2009).

All these differences signify that information about variation in diet flexibility and feeding histories is another interesting trait to follow in the species‐specific ecology of *Calanus* and is worth considering to understand how they individually utilize resources.

### 4.6 Ecological consequences

Rapid biogeographical shifts in plankton have already occurred in the North Atlantic Ocean (Beaugrand et al., 2009), and now, analogous processes are occurring in the Arctic (Wassmann, 2011). The central Arctic Ocean has the highest variability in climate and, therefore, the lowest predictability in food availability, both between years and seasons (Lewis et al., 2020; Polyakov et al., 2020), so it is an area with a high level of competition. Most likely, the crucial determinant, which would be the winner in the future Arctic ecosystem, is ecological plasticity, which is known to be remarkable in *Calanus* (Falk‐Petersen et al., 2009) but is not necessarily the same across species and environments.

An experimental study of the effects of temperature and food on *C. finmarchicus* and *C. glacialis* implies that the adaptation of *C. glacialis* to future warmer ocean conditions in the Arctic by spawning early will no longer be beneficial when *C. finmarchicus* matches the timing of gonad maturation with blooms (Kjellerup et al., 2012). Moreover, *C. glacialis* is probably not capable of exploiting longer periods of food availability in contrast to *C. finmarchicus* (Freese et al., 2016; Swalethorp et al., 2011). At the same time, the northward expansion of *C. glacialis* and its greater developmental rate are predicted to be an effect of increased primary production and decreased extent of ice in the central Arctic (Feng et al., 2018). While *C. finmarchicus* biomass and production may prevail in the future in northern regions due to its increased movement via advection, higher growth rate and shorter life cycle (Renaud et al., 2018; Scott et al., 2000; Weydmann, 2018), *C. glacialis* may also exhibit large spatial variability in its phenology, energy allocation, reproduction, and adaption to a wide range of environmental conditions (Banas et al., 2016; Bandara et al., 2019; Daase et al., 2013; Feng et al., 2018).

Our results show that the plasticity of both *Calanus* species is indeed impressive and may be differently applied, depending on the conditions in the water mass in which they reside, namely if they live in their “comfort zone” or beyond it. Our observations imply that when present at the same place and time, they can reach different sizes, take on different pigmentation, accomplish different states of population development, utilize different reproductive versus lipid accumulation strategies, and thrive on different foods. *C. finmarchicus* was mostly “the guest” in the studied subregimes and it showed much more flexibility in most of the studied ecological traits, which suggests that it has strong potential to colonize newly available Arctic habitats under progressing climate change. In agreement with our hypothesis, the extent of plasticity was higher in *C. finmarchicus*, which seems to be more of a generalist than *C. glacialis*. Nevertheless, models are optimistic not only for the future of *C. glacialis* (Feng et al., 2018) but also for *C. hyperboreus*, which is expected to survive, albeit by becoming more “boreal‐like” (Kvile et al., 2018). Looking at the uncertainties raised in our study, we suggest that the role of ecological plasticity is probably not only a matter related to the species but also to the population. Additionally, the question whether *Calanus* will ever reach a tipping point with regard to its high ecological plasticity, regardless of the species, remains unanswered, hopefully forever. It is also possible that *Calanus* will be able to adjust to climate change through adaptive responses, provided that evolutionary changes occur at a rate similar to climate change (Dam, 2013).

## CONFLICT OF INTEREST

None declared.

## AUTHOR CONTRIBUTION

**Emilia Trudnowska:** Conceptualization (lead); Data curation (lead); Funding acquisition (lead); Investigation (lead); Methodology (supporting); Project administration (lead); Resources (lead); Visualization (lead); Writing‐original draft (lead). **Kaja Balazy:** Data curation (equal); Formal analysis (equal); Writing‐review & editing (equal). **Joanna Stoń‐Egiert:** Data curation (equal); Formal analysis (equal). **Irina Smolina:** Data curation (equal); Formal analysis (equal); Methodology (equal); Writing‐review & editing (equal). **Thomas A. Brown:** Data curation (equal); Formal analysis (equal); Methodology (equal). **Marta Gluchowska:** Conceptualization (supporting); Funding acquisition (supporting); Investigation (supporting); Project administration (supporting); Writing‐review & editing (supporting).

## Supporting information

Table S1‐S2

## Data Availability

Data are publicly available in Dryad portal: https://doi.org/10.5061/dryad.x69p8czgs
