# Supplementary material for: In a comfort zone and beyond—Ecological plasticity of key marine mediators
Source: Ecol Evol. 2020 Nov 10;10(24):14067–81. doi: 10.1002/ece3.6997 (PMC7771121; doi:10.1002/ece3.6997)
Supplement: Supplementary file 1 — Table S1‐S2 [file ECE3-10-14067-s001.docx]

*Suppl. Table 1. The number of Calanus individuals selected for particular analyses at studied domains: AT (Atlantic), AR (Arctic), F (Fjord), G (Glacial bay).*

| Station/  Type of analysis | AT | AR | F | G |
| --- | --- | --- | --- | --- |
| Genetics | *C.* *fin* : 30 ind. | *C.* *fin* : 30 ind. *C.glac* : 30 ind. | *C.* *fin* : 30 ind. *C.glac* : 30 ind. | *C.* *fin* : 30 ind. *C.glac* : 30 ind. |
| Pigments (HPLC) | *C.* *fin* : 5 x 10 ind. | *C.* *fin* : 5 x 10 ind.  *C.* *glac* : 5 x 10 ind. | *C.* *fin* : 5 x 10 ind.  *C.* *glac* : 5 x 10 ind. | *C.* *fin* : 5 x 10 ind.  *C.* *glac* : 5 x 10 ind. |
| Lipid composition | *C.* *fin* : 5 x 10 ind. | *C.* *fin* : 5 x 10 ind.  *C.* *glac* : 5 x 10 ind. | *C.* *fin* : 5 x 10 ind.  *C.* *glac* : 5 x 10 ind. | *C.* *fin* : 5 x 10 ind.  *C.* *glac* : 5 x 10 ind. |
| Lipid content & image-based prosome lengths & pigmenatation | *C.* *fin* : 180 ind. | *C.* *fin* : 179 ind.  *C.* *glac* : 179 ind. | *C.* *fin* : 180 ind.  *C.* *glac* : 180 ind. | *C.* *fin* : 180 ind.  *C.* *glac* : 175 ind. |
| Net - based prosome lengths | 113 ind. | 109 ind. | 93 ind. | 57 ind. |
| Izotopes | *C.* *fin* : 5 x 10 ind. | *C.* *fin* : 5 x 10 ind.  *C.* *glac* : 5 x 10 ind. | *C.* *fin* : 5 x 10 ind.  *C.* *glac* : 5 x 10 ind. | *C.* *fin* : 5 x 10 ind.  *C.* *glac* : 5 x 10 ind. |
| Gonad stages | 397 ind. | 39 ind. | 37 ind. | 4 ind. |

*Supplementary Table 2. Relative composition (mean + standard deviation) of the trophic markers (signature according to* Lee, Hagen, & Kattner, 2006 and Leu et al, 2006) *derived from the fatty acids (FAs) composition in Calanus CV in various water domains.*

|  |  | AT | AR | | F | | G | |
| --- | --- | --- | --- | --- | --- | --- | --- | --- |
| FA | Marker | *C. fin* | *C. fin* | *C.glac* | *C. fin* | *C.glac* | *C. fin* | *C.glac* |
| C20:22 | MUFAs | 43±1.3 | 39±2.8 | 43±2.8 | 41±0.6 | 41±3.0 | 42±1.4 | 46±1.0 |
| C20:5n32 | EPA-Diatoms | 16±0.3 | 10±1.0 | 12±0.3 | 11±0.4 | 11±0.3 | 11±0.1 | 13±0.3 |
| C22:6n3 | DHA- dinoflagellate | 10±0.6 | 8±0.8 | 7±0.7 | 7±0.8 | 8±0.5 | 9±1.2 | 8±0.6 |
| C18:4n32 | Phaeocystis | 13±0.4 | 17±2.0 | 15±1.7 | 14±1.3 | 19±2.4 | 15±2.6 | 19±1.6 |
| C18:1n9 | Omnivory | 5±0.3 | 8±2.2 | 9±1.3 | 5±0.3 | 12±2.4 | 8±1.1 | 7±0.9 |
| C18:2n62 | Terrestial & freshwater | 0.26 | 0.34 | 0.18 | 0.24 | 0.26 | 0.20 | 0.24 |
| DHA/EPA | Flagellates / diatoms | 0.6 | 0.7 | 0.6 | 0.6 | 0.8 | 0.8 | 0.6 |
